# On Hydrocele

**Published:** 1843-12-09

**Authors:** 


### CLINICAL LECTURES AND REPORTS.

### PENNSYLVANIA HOSPITAL.

### CLINIC OF J. RANDOLPH, M. D.

[Reported for the Medical Examiner.]

*November* 25, 1843.

### LECTURE I.

### ON HYDROCELE.

Gentlemen:—I propose, to-day, to exhibit to you a patient affected with the complaint we term hydrocele. Previously I shall make a few remarks upon the nature of the disease, and point out to you the different methods of operating which have been resorted to for its cure.

The tunica vaginalis secretes in its natural state a limpid fluid, which lubricates its internal surface and those of the tunica albuginea; whenever this fluid from any cause is secreted in too great a proportion, it distends the tunica vaginalis, giving rise to a tumour of the scrotum, which is termed hydrocele. When the communication between the cavity of the abdomen and the tunica vaginalis is not closed as it should be at the usual time, the fluid descends from the cavity of the abdomen into the cavity of the tunica vaginalis, forming what is called a congenital hydrocele. Hydrocele is a disease of frequent occurrence; it is incidental to the young as well as the old; it is most commonly a local disorder; sometimes, however, it is combined with a hydropic condition of the system.

The causes which give rise to this disease are not well known. Mr. Pott says, that any cause which increases the natural secretion into the cavity of the tunica vaginalis, and prevents the absorbent vessels from taking up the fluid, will contribute to its formation. It may often be traced to external injury, such as blows, falls, strains and frictions. It is also owing sometimes to inflammation of the testis, and is frequently combined with stricture of the urethra, or local irritation along its course. Dr. Physick succeeded in curing a case of this disease by dilating the stricture by means of a bougie.

Hydrocele is sometimes conjoined with sarcocele, or chronic enlargement; in this case it is termed hydrosarcocele. It is very important to distinguish between these two diseases; and this distinction may be easily made by attending to the following circumstances; in sarcocele, the tumour is oval and flattened; it may attain a considerable size, without, however, ascending so near to the external abdominal ring as a large hydrocele does. In sarcocele there is a space between the top of this tumour and the abdominal ring, whereas, generally, there is none in a large hydrocele. The tumour may also be known by its weight and opacity. In hydrocele the swelling commences at the bottom; is generally confined to one side; at first the tumour is flaccid, and the testis may be felt through it; but as it increases in size it becomes firm and incompressible, and conceals the testis; the swelling assumes a pyriform shape, the corrugations of the scrotum disappear, and the raphe inclines to one side; generally there is little or no pain or inflammation; no alteration of colour. When inflammation, however, precedes this disease, there is pain, swelling, and hardness. The swelling in hydrocele is translucent, which, by the usual means, may be detected. In some cases the tunica vaginalis testis becomes thicker and harder, the fluid is opaque and dark coloured, thereby preventing the rays of light from passing through it. Under these circumstances there would be no transparency.

Sometimes an accumulation of fluid takes place in the tunica vaginalis of the spermatic cord, forming an encysted hydrocele of the spermatic cord. This disease may be confounded with hernia, from which it is very necessary to distinguish it. In hydrocele of the cord the accumulation takes place gradually, unattended with pain, and is always below the external abdominal ring. When the patient coughs there is no impetus communicated to the finger, and the tumour is not capable of being returned into the cavity of the abdomen; whilst in hernia, the swelling takes place suddenly, after some violent effort, attended with pain; and the peculiar impetus is communicated from the tumour when the patient coughs, and it may generally be returned by pressure into the abdomen. When both these diseases take place in the same person, the diagnosis is difficult. The scrotum is liable to anasarcous swellings, which is termed anasarcous tumour of the scrotum, or hydrocele œdematodes. In this case the scrotum is swelled on both sides; serum or lymph is effused into the cellular tissue, exterior to the tunica vaginalis testis; the indentations of the fingers remain after pressure, and there is generally a hydropic condition. The treatment consists of general and local remedies. The local means consist in puncture and compressure. In young children this disease may frequently be cured by absorption; to this end the patient should be purged by small doses of calomel and rhubarb, or cremor tartar and jalap, and the scrotum supported by a suspensory bag, and kept wet with a solution of muriate of ammonia, ʒij., in liq. acet. am. ℥vj., as advised by Sir A. Cooper. The affusion of cold water, and the muriate of ammonia dissolved in vinegar and laudanum, may also be used with advantage; but this treatment will not succeed in adults. The treatment in these cases is divided into palliative and radical. The palliative consists in the operation of tapping the tunica vaginalis testis, and letting the fluid escape. The scrotum should be placed in a suspensory bag, and pressure should be employed. The radical treatment consists in exciting inflammation, so as to bring about adhesion between the tunica vaginalis testis and tunica albuginea, closing the cavity of the bag, and preventing a receptacle for the water.

It is generally proper to resort to the palliative treatment, and to ascertain the condition of the testis, before attempting the radical cure. It is necessary to guard against excessive inflammation. Tapping has been known sometimes to produce a permanent cure, but this event is rare. The most ancient operation was that described by Celsus, which consists in making an incision into the tunica vaginalis testis to the bottom of swelling, and introducing lint. The objection to this is the danger of too great inflammation. The second method consists in excising a portion of the tunica vaginalis testis, which also is objectionable, from the excessive inflammation it may produce. The third method consists in applying vegetable caustic, causing sloughing of the tunica vaginalis; but here the cure is tedious, and accompanied by troublesome ulceration. This mode of operating was advocated by Wiseman. Another mode is to introduce a tent of sponge or lint, or a canula, or short piece of gum elastic catheter, as advised by Baron Larrey into the tunica vaginalis testis: there is in this danger of injuring the testicle. The introduction of a seton has also been frequently employed, and was particularly recommended by Mr. Pott.

The last method consists in injecting some stimulating fluid into the cavity of the tunica vaginalis, so as to bring about the necessary inflammation. For this purpose port wine has been used, in the proportion of two-thirds wine to one-third water. A solution of sulph. zinci has been tried; iodine, cold water, the fluid drawn off, have all been used; but port wine is considered by Dr. Randolph to be the best.

The length of time that the liquid shonld remain has been found to be about five minutes; but in textures, which are very thin and vascular, two or three minutes have been found long enough. In some cases port wine has been injected undiluted. It is requisite to keep the extremity of the canula well within the tunica vaginalis testis, to prevent the fluid from escaping into the cellular tissue, and producing inflammation and sloughing. If the inflammation runs too high, it is necessary to put the patient on antiphlogistic treatment; purgatives, low diet, rest, and cold applications should be enjoined. If the inflammation is not sufficiently great, cause the patient to walk about, give him good di[illegible]t, wine. The after treatment might depend on the degree of inflammation.

The man was now introduced into the amphitheatre, and Dr. Randolph drew off the fluid by puncturing the tunica vaginalis by means of a trochar. The disease first made its appearance in October last a year, and supervened three or four weeks after a fall.

